# Machine unlearning as a governance imperative for clinical AI

**DOI:** 10.1038/s41746-026-03050-1

**Published:** 2026-07-18

**Authors:** Anthony Porter, Emily Kirkpatrick, Arpit Garg, Hemanth Saratchandran, Simon Lucey, Johan Verjans

**Affiliations:** 1 Australian Institute for Machine Learning (AIML), The University of Adelaide, Adelaide, SA Australia; 2 Central Adelaide Local Health Network (CALHN), Adelaide, SA Australia; 3 South Australian Health and Medical Research Institute (SAHMRI), Adelaide, SA Australia; 4 Jones Radiology, Adelaide, SA Australia

**Keywords:** Computational biology and bioinformatics, Health care, Medical research, Scientific community

## Abstract

Clinical AI assumes that the influence of training data can persist indefinitely. This premise fails when patients withdraw consent, evidence evolves, or bias is identified. Machine unlearning aims to remove specific data influence without full retraining. We argue that unlearning readiness should be built into the infrastructure of high-risk healthcare AI across patient autonomy, clinical validity, and system governance, and we outline a governance pathway to keep updates auditable and clinically safe.

## Introduction

Healthcare AI currently rests on a fragile assumption: once an algorithm has “learned” from data, that knowledge may remain valid and usable indefinitely. In practice, this assumption breaks down quickly: patients invoke data-protection rights following breaches or withdrawal of consent; permitted uses change; clinical evidence evolves; and long-standing biases are exposed in historical datasets. As seen during the rapid turnover of COVID-19 treatment guidance, models trained on yesterday’s evidence can become unsafe within months^1^. At the same time, well-documented racial and other inequities are embedded in datasets used for risk prediction and resource allocation^2–4^.

Clinical AI is increasingly deployed under continuous improvement models, with iterative updates, recalibration, and model replacement occurring within active clinical workflows. In parallel, health systems are scaling multi-site and federated deployments in which data rights and consent conditions can vary across institutions and change over time. Patient withdrawal is therefore not an exceptional event but a foreseeable lifecycle scenario, particularly for models trained on large longitudinal datasets. Regulators and hospital boards are also moving toward lifecycle governance expectations that emphasise traceable updates, auditable change control, and post-change evidence of safety and performance. Digital medicine turns unlearning from a theoretical concern into a practical governance question. Machine unlearning refers to methods that modify a trained model so its behaviour approximates (or matches) a counterfactual model trained without specified data^5^. We distinguish unlearning of model parameters from deletion in retrieval-augmented systems (where patient data may not be embedded in weights) and from full retraining.

These pressures collide with the way most clinical AI is actually built. Once deployed, there is rarely a safe way to remove the influence of specific patients, sites, or subgroups without rebuilding the system: deleting a record from a database does not erase its imprint on model parameters or downstream services. For patients, this raises questions about how rights to erasure and data sovereignty can be honoured when source-data deletion does not propagate to model behaviour^6^; for clinicians, it threatens calibration and fairness when knowledge changes; for health systems, it complicates lifecycle governance under the General Data Protection Regulation (GDPR)^7^, the EU AI Act^8^, and emerging device guidance such as the U.S. Food and Drug Administration (FDA)’s Predetermined Change Control Plan (PCCP)^9^. Whether Article 17 erasure obligations extend to trained model parameters remains legally untested, though regulators have begun to address the question: the European Data Protection Board’s Opinion 28/2024 emphasises case-by-case assessment of when AI models still involve processing personal data^10^, and the UK Information Commissioner’s Office notes that individual rights may apply even where personal data may be contained in the model itself^11^.

Machine unlearning offers one of the few plausible technical routes to address this permanence problem^5^. However, it is rarely specified in procurement, seldom supported in deployed systems, and largely absent from clinical AI governance frameworks. Existing work has made the ethical case for unlearning and surveyed technical approaches in digital health^12,13^. What is missing is a translation into concrete decisions for health services and regulators.

The pressure for unlearning increases as healthcare adopts foundation models and “clinical copilots,” which are often vendor-hosted and updated continuously through iterative fine-tuning, retrieval updates, and safety patches only partially visible to deploying organisations. They may also learn from mixed data streams beyond the electronic health record (EHR), including free-text notes, imaging, and wearable remote monitoring data, where withdrawal requests or bias findings may span heterogeneous sources with different consent conditions and governance owners. Organisations therefore need auditable evidence that the influence of specified data has been removed and clinical performance remains safe after each update.

In this Perspective, we argue that high-risk clinical AI should be designed so that targeted remediation of learned data influence is feasible where legal, ethical, community-governance, or clinical-safety grounds require removal or correction. Our claim is forward-looking: systems built without any capability for targeted remediation are likely to become difficult to reconcile with emerging expectations for data rights, lifecycle governance, auditability, and clinical safety. We frame unlearning at three stakeholder levels (patients, clinicians, and health systems) and show how it can be integrated into routine model lifecycle management rather than treated as a rare, heroic intervention. We connect individual rights (including Indigenous data sovereignty and the CARE principles)^14^, GDPR erasure rights^15^, and emerging AI lifecycle regulation (including the EU AI Act^8^ and FDA PCCP guidance^9^) with practical patterns for governance, infrastructure, and evaluation. Our aim is not to provide a comprehensive technical survey but to outline what it would take for health services to make machine unlearning a functioning part of clinical AI infrastructure.

## When might machine unlearning be needed?

Several types of trigger may call for remediation by machine unlearning, with different legal, ethical, and clinical bases. Machine unlearning is most relevant where the concern is not only the retention or future use of source data, but the continued influence of specified data on a trained model.

### Patient withdrawal or erasure requests

A patient may withdraw from a longitudinal registry, biobank, or hospital data programme used to train a clinical model. Source records may need to be deleted, restricted, or excluded from future use; whether that obligation extends to the model’s learned behaviour is a separate and context-dependent question. Some regimes are deliberately prospective. For example, the European Health Data Space’s Article 71 opt-out applies to future secondary use rather than retrospective removal from already trained models, reflecting in part the scientific and operational difficulty of tracing and removing data contributions after model development^16^.

### Indigenous data sovereignty and community-governed withdrawal

Unlearning may also be triggered by collective governance obligations rather than individual data-protection rights. An Aboriginal and Torres Strait Islander community might withdraw approval for use of community-governed data in a diabetes-risk model. The relevant duty may arise from a research agreement, ethics approval, fiduciary relationship, community protocol, or Indigenous data-governance framework. In such cases, remediation may need to address not only source-data access but also the model-level influence of a community, cohort, site, or dataset.

### Lapsed licences, provenance failures, data poisoning, or discovery of bias

The basis for using particular data may change after deployment. A licence for an external imaging dataset may expire; provenance checks may reveal that records were used outside their permitted scope; an attacker may have injected poisoned wearable-device observations; or a post-hoc audit may show that a model has learned a harmful proxy from a site-specific coding practice. In each case, continued reliance on the learned contribution of the affected data may be inappropriate even where the source data can be removed, quarantined, or excluded from future training.

### Clinical-safety remediation

Unlearning may also be part of a clinical-safety response. A sepsis model may have been trained on outcome labels later found to be erroneous; a deterioration model may have learned from corrupted sensor streams; or a radiology model may have incorporated artefacts from a defective annotation process. The appropriate response may include relabelling, feature masking, recalibration, retraining, or selective unlearning. These steps are not interchangeable: relabelling or masking may correct the data pipeline, while retraining or unlearning addresses the model’s learned dependence on the affected data. In practice, clinical remediation may require both.

The relevant removal target also varies in granularity. A patient may contribute longitudinal, multimodal data, so the practical question is often whether the affected unit is a whole patient record, a single encounter, a site, a cohort, a modality, a feature, an annotation, or a corrupted data stream. We use *record-level unlearning* for removal of the learned influence of a whole patient, case, site, or cohort, and *attribute- or feature-level unlearning* for removal of the learned influence of a specified feature, modality, annotation, or episode. This distinction matters because the governance trigger and technical remedy may differ: withdrawal or licence failure may point toward patient-, site-, dataset-, or cohort-level unlearning, whereas erroneous labels, biased features, or corrupted measurements may first require correction, masking, recalibration, or retraining on corrected data.

## Three stakeholder levels for unlearning governance

### For patients: sovereignty, rights and autonomy

Consider a fictionalised scenario derived from real events. An Indigenous person discovers that de-identified health data have been used to train a diabetes complications prediction model without ongoing, culturally informed consent. They request withdrawal, citing Indigenous data sovereignty^14^. Even with de-identification, re-identification risk and community governance expectations can justify model-level remediation. The hospital wishes to honour the request, but the AI developer explains that deleting a single patient’s data ordinarily requires full retraining at substantial cost and downtime. The choice becomes stark: decline the request, risking breach of community-governance commitments and leaving unresolved data-rights questions, or rebuild the model.

Similar dilemmas arise where GDPR erasure rights apply to patients’ personal data^15^, or where young people wish to “reset” their data footprint when transitioning from paediatric to adult services. In these cases, data withdrawal is not only an individual right but also a collective governance question for communities and cohorts. Long-running registries and biobanks must accommodate withdrawals without collapsing under the cost of repeated full retraining.

When combined with Indigenous data sovereignty and the CARE principles^14^, unlearning supports community-level control over how data are used, withdrawn, and audited over time.

### For clinicians: quality and safety

Clinicians increasingly see AI outputs alongside routine investigations and risk scores^17^. From their perspective, the central question is whether patient data can be withdrawn or relabelled without silently degrading model performance, especially in vulnerable subgroups.

Heart failure illustrates this problem. Evolving classifications based on ejection fraction and changing thresholds mean that labels applied a decade ago may no longer reflect current definitions^18^. Decision-support tools trained on such data risk embedding obsolete knowledge into present-day care unless those outdated labels are corrected, relabelled, or masked, or the affected model is recalibrated, retrained, or selectively unlearned. Similar issues arise when tumour taxonomies change, laboratory reference ranges are updated, or practice guidelines are reversed.

For clinicians, unlearning events should resemble any other regulated model update rather than a mysterious back-end process. There should be clear documentation of what changed and why, pre-specified non-inferiority thresholds, subgroup performance checks, and a governance body accountable for sign-off. Integrating unlearning into existing clinical change-control processes is far preferable to treating it as a rare, purely technical operation managed solely by data scientists.

### For systems: adaptability and governance

Health services face competing pressures to adapt AI as data, evidence, and regulations change, while maintaining traceability and safety^8,9^. In principle, machine unlearning allows a system to remove specific influences (an individual, a site, a poisoned cohort) while keeping models within acceptable performance margins. In practice this only works if unlearning is treated as a clinical change with governance.

Organisations should define a small number of clear triggers (patient or community withdrawal, data licence lapses, audit-flagged bias or poisoning, and substantial guideline or labelling changes) and pre-register a validation plan for each, specifying which metrics and subgroups must be re-checked and what non-inferiority margins apply. Failed validation should trigger rollback and escalation to a clinical AI safety committee.

None of this is possible without basic infrastructure: a model registry, robust data lineage, versioned datasets and models, and append-only audit logs. Clinician-facing revision notes in EHRs should explain what changed, why, and with what effect on performance and calibration.

These challenges differ by architecture. For closed-source, vendor-hosted models, deploying organisations have no access to weights and might require attestations or technical evidence from AI vendors that models can honour data deletion requests, as part of procurement or approval. For fine-tuned foundation models, unlearning addresses only the fine-tuning contribution, not what the base model learned. Retrieval-augmented systems offer a more tractable alternative: patient data remains in organisation-controlled stores, and unlearning becomes largely document deletion rather than model modification. Where clinical performance permits, retrieval-augmented architectures may therefore be preferable for consent-sensitive or frequently changing patient data, though they do not eliminate all safety or privacy risks. Prompt histories, retrieval logs, any fine-tuning data, and the base model itself may still remain in scope for privacy, safety, or governance review.

### Reality check: technical approaches and tradeoffs

The cleanest way to remove data influence is to retrain from scratch on a dataset with the target records removed^5^. This “exact unlearning” offers the strongest guarantee that model behaviour matches a counterfactual without the data, but it can require days or weeks of computation on specialised hardware and substantially increase computational cost, carbon footprint, and downtime. Exact unlearning will remain a gold standard against which more practical methods are judged.

Most current work focuses on approximate unlearning methods that mimic full retraining at lower cost^19^. These include influence-estimation techniques that adjust the contribution of specific points, and partitioned or ensemble training (e.g., sharded subsets) that localises unlearning to affected components. The tradeoff is design complexity and potential accuracy loss, which may be acceptable in high-volume systems if safety remains within predefined margins.

Certified methods provide formal guarantees, often building on differential privacy, that an unlearned model is statistically indistinguishable from one retrained without the forgotten data^20^. These guarantees are mostly available for simpler model families; scalable certified unlearning for the deep architectures common in imaging and complex EHR modelling remains largely experimental and unrealistic as a near-term regulatory baseline^21^.

As a pragmatic decision rule, small models, and high-risk models where deletion volume and operational constraints permit, should default to full retraining; systems expecting frequent or large-scale deletion should be designed for partitioning from the outset (e.g., sharding) to keep repeated unlearning operationally feasible. Where strong removal guarantees are required, certified methods should be used where feasible; otherwise stricter post-unlearning validation, documentation, and explicit acceptance thresholds should be used as compensating safeguards.

Whether approximate methods can satisfy ethical or legal requirements depends on the trigger. Exact retraining remains the strongest benchmark; approximate methods are best understood as risk-reduction tools and may be inadequate where the trigger is consent-driven privacy erasure, in which case certified methods, full retraining, or model retirement may be required. This is not equivalent to asking whether de-identification is sufficient: de-identification operates on raw records and does not change what a model has already learned from them, whereas approximate unlearning operates on the model itself and attenuates learned influence after training. The two address different objects, and the assurance each provides depends on the threat model, the strength of the unlearning method, and the residual signal that can be recovered from the modified model. In practice, deployed examples of machine unlearning remain limited; the clearest are concept-erasure techniques used in commercial text-to-image diffusion models (for example, removal of specific artist styles or adult content from Stable Diffusion deployments)^22^ and federated unlearning of withdrawn-site contributions in collaborative medical imaging research^23^.

Table 1 summarises the main unlearning strategies, their privacy properties, computational cost, and implementation complexity. In practice, the key question for governance is not whether unlearning is mathematically perfect, but whether it delivers sufficiently aligned behaviour under robust validation to satisfy legal and clinical safety obligations.

**Table 1:** Comparative analysis of machine unlearning approaches in healthcare

Approach	Removal assurance	Computational cost	Implementation complexity	Best clinical use cases
Exact unlearning/full retraining	Strongest practical guarantee: model behaviour matches a model trained on the remaining data only	Prohibitive for large models: requires full retraining, often taking days to weeks	Low conceptual complexity: no architectural changes required	Small models; rare high-stakes erasure requests where strongest removal assurance is required and retraining is feasible
Approximate unlearning/influence-based methods	Probabilistic: aims to minimise residual influence under validated conditions	Moderate: additional parameter updates, often hours	Moderate: requires influence or gradient estimation	Clinical documentation and summarisation tools; large EHR-based risk prediction models
Data partitioning/SISA-style training	Localised removal: supports targeted removal for data in the affected shard or partition	Low to moderate: retraining limited to the relevant partition	Moderate: requires planned data sharding and additional storage from model development	High-volume systems with frequent withdrawal requests, such as wearables or remote monitoring
Certified unlearning/often DP-based	Formal guarantee: statistical bounds on residual influence, often derived from differential privacy	High: DP-aware or certified training may reduce utility	High: specialised pipelines required; currently feasible mainly for simpler models	Highly sensitive datasets, such as genomics or HIV registries, under strict regulatory scrutiny; mostly emerging in practice

The table summarises four principal strategies for removing patient data influence from trained AI models, comparing their privacy guarantees, computational cost, and implementation complexity. The “Best clinical use cases” column provides pragmatic examples of where each approach may be most appropriate. Exact unlearning offers the strongest practical privacy guarantee but is rarely feasible for large models. Approximate unlearning provides operationally viable alternatives for many clinical applications, but its assurance is probabilistic and validation-dependent. Partition-based and certified approaches require design-time architectural decisions. Clinical use cases reflect expert judgement rather than empirically validated pairings.

*SISA* Sharded, Isolated, Sliced, Aggregated training, *DP* differential privacy.

## Barriers to implementation

### Clinical safety and performance preservation

When a model is asked to remove the influence of a patient’s, site’s, or cohort’s data, it can sometimes also lose important knowledge that was meant to be retained. This unintended loss, analogous to the ‘catastrophic forgetting’ phenomenon described in continual learning, can occur during the unlearning process itself^24^. For example, a dermatology AI that unlearns melanoma cases from one group may underperform for patients of similar skin types, creating a clinical safety risk. Robust safeguards are therefore needed to ensure that removing one patient’s data does not unintentionally degrade the model’s broader clinical performance.

### Privacy failures and incomplete deletion

Unlearning can also fail in the opposite direction: models may retain substantial information about supposedly forgotten data. In a collaborative prostate segmentation setting, naive fine-tuning approaches preserved a large fraction of diagnostic performance on data that were meant to be removed, whereas full retraining reduced that performance substantially^23^. That gap represents not just a technical flaw but a failure to honour data withdrawal.

Effective remediation requires propagation across the full pipeline, encompassing raw data, model versions, and all downstream services^25^. If any node is missed, a residual signal persists. In federated networks, the complexity multiplies because organisations must determine which sites unlearn, in what sequence, and how the remaining sites resynchronise. Most health systems lack robust data-lineage tooling even for conventional analytics^26^. Without this, they cannot credibly demonstrate that unlearning requests have been fully honoured^27^.

### Computational and regulatory gaps

Exact unlearning often consumes weeks of processing for large models, while approximate methods introduce uncertainty about residual influence under large forget sets. Some privacy regimes grant withdrawal or erasure rights but remain vague about adequate erasure in complex AI pipelines. The EU AI Act and FDA Predetermined Change Control Plan guidance set general requirements (traceability, logging, risk management, pre-specified triggers and acceptance criteria) that arguably extend to unlearning^8,9^. But neither provides concrete standards for demonstrating effective unlearning without unsafe drift.

### Global equity and two-tier standards

The equity implications of unlearning cut both ways. There is a real risk of a two-tier ecosystem in which well-resourced health systems develop and enforce strong expectations for unlearning, while models without feasible targeted-remediation capability are exported to more resource-constrained settings that lack the infrastructure to demand equivalent safeguards. In such settings, patients may have formal data rights on paper but no realistic way for those rights to affect deployed models, entrenching disparities in whose data can be revoked and whose biases can be corrected. Conversely, blanket mandates that ignore local capacity could push AI access out of reach for the same communities, particularly where shared infrastructure for verification or retraining is unavailable. Unlearning requirements should therefore be proportionate, risk-based, and supported by shared infrastructure. International procurement templates, shared assurance test batteries, and open reference implementations could help avoid both outcomes.

## Pathways forward

Health services face four main problems when making unlearning routine: unclear governance, fragile technical plumbing, immature methods, and uncertain evaluation standards. Box 1 summarises minimum procurement requirements for unlearning-ready clinical AI.

First, governance. A designated authority must decide when a deletion request is legitimate, which models and downstream services it affects, and what performance degradation is tolerable. Organisations should define a clear chain of responsibility (e.g., Clinical AI Safety Lead plus designated committee) and written criteria for when unlearning is triggered and what evidence is required. Box 2 summarises an implementable governance pathway.

Second, infrastructure. Without robust data lineage, versioned models, and a model registry, unlearning will remain a theoretical aspiration. Teams must track where each training example influences deployed models, maintain version control for models and datasets, and reserve compute for deletion and re-validation.

Third, method selection. Not every model needs the same strategy. Small models, and high-risk models with limited data, may justify full retraining; large EHR models may require approximate or partition-based methods; rare disease or genomics applications may suit certified or near-certified approaches as they mature.

Finally, evaluation. There is no agreed “unlearning test set”. A pragmatic interim solution is a core battery of checks (discrimination, calibration, key subgroups, membership inference where feasible) re-run after each event, with thresholds pre-specifying both absolute floors and tolerable relative degradation. Emerging frameworks (ISO/IEC 42001:2023 and NIST AI RMF 1.0) provide governance templates; healthcare-specific unlearning benchmarks remain undeveloped^28,29^. Where assurance thresholds cannot be met, model disgorgement may be the only defensible remedy^30^.

Box 1 Minimum procurement requirements for unlearning-ready clinical AI systems

- Traceability: dataset manifest, consent and licence status, and lineage to model versions.
- Model registry: versioned artefacts and configuration, validation reports, and rollback capability.
- Triggers: withdrawal, licence lapse, bias or poisoning findings, and guideline or label changes.
- Declared approach: retraining, approximate unlearning, partitioned unlearning, or certified unlearning, with an assurance target.
- Validation: discrimination, calibration, subgroup and fairness checks, and privacy tests where feasible.
- Audit and communication: append-only logs and clinician-facing release notes.
- Operational service levels: timelines, acceptance thresholds, rollback criteria, and escalation pathways.

Box 2 Governance pathway for unlearning in deployed clinical AI

1. Intake: log the request, verify authority, and define scope and trigger.
2. Impact mapping: use the model registry and data-lineage records to identify affected models, versions, and services.
3. Containment: decide whether to continue, restrict, roll back, or suspend deployment pending remediation.
4. Approval: set the required assurance level, acceptable downtime, and validation margins.
5. Method choice: select retraining, approximate unlearning, partitioned unlearning, or certified unlearning; document the rationale.
6. Execution: lock the baseline, perform unlearning in a controlled environment, and record artefacts and forget targets.
7. Validation: run the pre-specified validation battery; if thresholds fail, roll back or escalate.
8. Deployment: update the registry and audit log, publish release notes, monitor drift, and close the case.

## Conclusion

Machine unlearning is an important technique for remediating high-risk clinical AI, applicable where patient rights, community governance, data integrity, or clinical safety require removal or correction of learned influence. It is not a universal legal requirement for all clinical AI. When consent is withdrawn, evidence changes, or bias is identified, organisations need a credible remediation pathway with traceability and clinical oversight. Preparation requires clear triggers, accountable decision-making, pre-specified validation (including subgroup checks where relevant), auditable change logs, and a defined rollback or retirement pathway if thresholds are not met.

Regulators can accelerate progress by clarifying what constitutes adequate erasure in complex AI pipelines and specifying minimum expectations for post-remediation assurance. Developers and vendors should design systems so that targeted remediation and verification are operationally feasible rather than exceptional. The question is not whether unlearning will be requested, but whether health systems will be equipped to manage it as a controlled lifecycle event rather than a disruptive response to external pressure.

## Data Availability

Data sharing is not applicable to this article as no datasets were generated or analysed during the current study.
